# Shear Wave Elastography in Children: Normative Values and the Impact of Healthy Habits in a Cross‐Sectional Study

**DOI:** 10.1002/hsr2.72653

**Published:** 2026-06-19

**Authors:** Dolors Casellas‐Vidal, Raquel Font‐Lladó, Inés Osiniri, Maria Camós‐Carreras, Aintzane Ruiz‐Eizmendi, Juan Serrano‐Ferrer, Joaquim Casellas, Abel López‐Bermejo, Anna Prats‐Puig

**Affiliations:** ^1^ Servei de Pediatria Hospital Universitari Doctor Josep Trueta Girona Spain; ^2^ Neurodevelopmental Group [Girona Biomedical Research Institute]‐IDIBGI Institute of Health Assistance (IAS), Parc Hospitalari Martí i Julià Girona Spain; ^3^ University School of Health and Sport (EUSES) University of Girona Girona Spain; ^4^ Research Group of Culture and Education, Institute of Educational Research University of Girona Girona Spain; ^5^ Servei de Pediatria, Clínica Bofill Girona Spain; ^6^ Servei de Rehabilitació Hospital Universitari Doctor Josep Trueta Girona Spain; ^7^ Departament de Ciència Animal i dels Aliments Universitat Autònoma de Barcelona Bellaterra Spain; ^8^ Pediatric Endocrinology Group Girona Biomedical Research Institute (IDIBGI) Salt Spain; ^9^ Research Group of Health and Health Care, Nursing Department University of Girona Girona Spain

**Keywords:** children, healthy habits, lifestyle factors, muscle elasticity, shear wave elastography

## Abstract

**Objectives:**

Biomechanical properties of the musculoskeletal system are important to understand muscle function, but they are difficult to assess noninvasively. Shear wave elastography (SWE) allows objective quantification of muscle elasticity. However, normative data and the influence of lifestyle habits on muscle elasticity in children remain unclear, particularly across multiple upper and lower limb muscles and in relation to modifiable healthy behaviors. The main objective was to assess muscle elasticity at rest and during maximum passive stretching (MPS) in children using SWE, and to address existing gaps by evaluating multiple muscles from both limbs, as well as to evaluate the influence of lifestyle factors, with particular attention to healthy habits.

**Design and Methods:**

SWE was used to measure the elasticity of the biceps brachii, pronator teres, adductor longus, lateral gastrocnemius, and soleus in 44 typically developing children (mean age 8.9 years; range 3–14 years; 27 females). Measurements were taken at rest and MPS. Data included anthropometry, physical fitness, Mediterranean diet adherence, moderate‐to‐vigorous physical activity (MVPA), and sleep duration, allowing the exploration of lifestyle‐related determinants of muscle elasticity.

**Results:**

Reliability estimates exceeded 0.91, indicating excellent consistency. SWE values increased with higher Mediterranean adherence during MPS in all muscles, while at rest, an inverse relationship appeared in biceps and pronator teres. Sleep time was negatively associated with SWE at rest and positively adductor longus during MPS. MVPA showed some associations without consistent patterns.

**Conclusions:**

SWE is a reliable and objective technique to evaluate muscle elasticity in typically developing children. By providing multi‐muscle reference data from both upper and lower limbs and demonstrating associations with diet and sleep, this study extends current pediatric SWE literature beyond feasibility and reliability. These findings suggest that SWE could help quantify the physiological impact of healthy habits on muscle properties during childhood.

## Summary

- SWE demonstrated high intra‐operator reliability for muscle elasticity assessment in children.
- Muscle elasticity differed between rest and stretch conditions, with greater uniformity observed during MPS.
- MD adherence and longer sleep time were positively associated with specific SWE patterns.
- No significant associations were found with age, sex, BMI, or physical activity level.
- The inclusion of five muscles from both upper and lower limbs provides a robust reference for pediatric SWE applications.

**Abbreviations**
AL: adductor longus
BB: biceps brachii
BMI: body mass index
ICC: intraclass correlation coefficient
LG: lateral gastrocnemius
MD: Mediterranean diet
MPS: maximum passive stretching
MVPA: moderate to vigorous physical activity
PT: pronator teres
ROM: range of motion
SOL: soleus
SWE: shear wave elastography
TD: typically developing

## 1 Objectives

Biomechanical properties of the musculoskeletal system are important to understand muscle function, but they are difficult to assess [1, 2]. Strength, joint range of motion (ROM), and muscular elasticity are important factors predicting physical function [3]. Strength and joint ROM are collected in current clinical practice. However, muscle elasticity is inferred by determining the muscle resistance moving the joint through its full range of movement, with some subjectivity [3, 4]. On the other hand, isolating muscle elasticity from tendons or joint capsular influences on joint movement by physical exam results unfeasible [4].

Adequate nutrition, physical activity, and sleep are among most determinant habits of a healthy lifestyle, and over the past decade an increasing number of studies have highlighted benefits on child musculoskeletal fitness [5, 6, 7, 8, 9, 10, 11]. The Mediterranean diet (MD) has been accepted as one of the healthiest dietary models [12], and associated with better physical and mental health (13,14, as well as strength improvements in children [14] and young adults [15]. More time spent in physical activity and sleep were associated with strength [9]. So far, the effect of a healthy lifestyle on muscle characteristics has not been studied.

The recent development of ultrasound techniques such as shear wave elastography (SWE) allows for a direct quantification of the speed of deformity of the muscle tissue in children and adults [3, 4, 5, 6, 7, 8, 9, 10, 11, 12, 13, 14, 15, 16, 17, 18] when a mechanical stress is applied [19, 20]. There are limited studies evaluating muscular elasticity in children, and factors influencing them; especially, contributions from healthy habits remain unknown. Moreover, all studies focused on one or two muscles from the same limb, mostly from the lower limb [3, 21, 22, 23, 24]. An accurate elasticity quantification grants a better knowledge of muscle properties and becomes mandatory to extend this information to multiple muscles from both upper and lower limbs at different stretching positions. Furthermore, factors influencing muscles properties, especially those modifiable as healthy habits, should provide essential information to predict and improve physical functions.

The main targets of this study were to assess reliability of SWE in children and to characterize muscular elasticity by SWE in typically developing (TD) children, in a total of five muscles from upper and lower limbs (biceps brachii [BB], pronator teres [PT], adductor longus [AL], lateral gastrocnemius [LG], and soleus [SOL] muscles). Moreover, factors influencing SWE were evaluated with a special emphasis on healthy habits (adequate nutrition, physical activity, and sleep).

## 2 Design and Methods

Of the eligible children, 44 TD children (average age 8.9 years; 27 female, 17 male) aged 3–14 were included in this study. Participants were recruited using a convenience sampling strategy from primary schools included in the physical education, health and children (PEHC) project [25]. Children were eligible if they had no history of neurological, musculoskeletal, or systemic conditions that could compromise physical function. This recruitment strategy and inclusion criteria were consistent with those used in our previously published work employing the same SWE methodology [26]. Potential selection bias was minimized by applying consistent inclusion criteria across all participants.

Parents or legal guardians of all children signed the informed consent to participate in the study. Approval by Hospital Josep Trueta Clinical Investigation Ethics Committee was also obtained (CEIC V4_14/11/2016).

This was a cross‐sectional observational study employing the methodological approach in our previous work [26] on muscle stiffness assessment in children using SWE, including probe positioning, acquisition settings, and ROI measurements. Briefly, SWE was performed by the same experienced pediatric echographist on five muscles—two from the upper limb (BB and PT) and three from the lower limb (AL, LG, and SOL)—using a GE Healthcare LOGIQ S8 XDClear ultrasound system with a GE ML6‐15‐D linear transducer. Measurements were taken at rest and during maximum passive stretching (MPS), following standardized protocols [24, 27], as detailed in Casellas‐Vidal et al. [26]. All SWE measurements were performed by the same trained operator, experienced in pediatric musculoskeletal ultrasound, to minimize interoperator variability. During MPS, only joint positioning was individualized for each participant and muscle, rather than fixed to predefined angles. MPS was defined as the end‐range passive joint position, determined by the onset of examiner‐perceived resistance or by the child's verbal or nonverbal expression of discomfort. This approach was adopted to account for interindividual variability in joint mobility and muscle‐tendon properties in children, and is consistent with previously published pediatric SWE protocols using the same methodology. For each muscle and position, five consecutive SWE measurements were obtained, and the arithmetic mean of these five values was calculated in accordance with the approach proposed by Lallemant‐Dudek et al. [28], who reported good to excellent reliability (intraclass correlation coefficient [ICC] > 0.7), in line with earlier findings [23, 26, 27].

Nutritional habits were collected by parental Kidmed questionnaire of adherence to Mediterranean Diet (0 to 10 points) [29]. Moderate to vigorous physical activity (MVPA) (min/day) and sleep time (hours/day) were measured with accelerometers (ActiGraph GT3X, Actigraph Corporation, Pensacola, FL) around children's waist for seven consecutive days (24 h/day). A compliant recording required at least 4 days, including 1 weekend day. For aquatic activities and showering/bathing, children were asked to remove the device. Accelerometers were programmed in epochs of one second and data were analysed using ActiLife [30] (see Table 1 for a summary of collected data).

The remaining data were collected the same day as SWE measurements (Table 1). Parents filled a questionnaire regarding gender, perinatological antecedents—gestational age (years), birth weight (g), and length (cm)—and age (years). A trained operator registered child weight (Tanita RD545, Tanita Europe BV, Amsterdam, The Netherlands) and height (stadiometer SE206, SECA, Hamburg, Germany) adjusted by age and sex standard deviation scores [31], waist (umbilical level), and calf perimeter (muscular bulk) at standing position by a measuring tape. Upper‐body muscular strength was measured twice at each hand (the highest value was retained). Children squeezed an analogic dynamometer (TKK 5001, Grip‐A, Takei, Tokyo) for at least 5 s at strength (grip span, 5 cm). Flexibility was assessed twice (the longest mark was retained) by sit‐and‐reach test with a standardized box (Baseline 12‐1085, Fabrication Enterprises Inc., New York, NY, USA) [32]. Joint ROM was measured as the difference between joint angle at rest and at MPS. This was measured using a Plurimeter Dr Rippstein (Medidevice, Desimed GmbH & Co., Bandenweiler, Germany) for the LG and SOL muscles and a goniometer (Baseline 12‐1006, Fabrication Enterprises Inc., NY, USA) for the other muscles.

**Table 1:** Lifestyle, demographic, anthropometric, and physical fitness characteristics of typically developing children by gender. Values are expressed as mean (range). Units are reported in the table.

	Female	Male
MVPA, min/day	55.8^a^ (22.3–89)	76.2^a^ (24.7–168.2)
Mediterranean diet adherence, 0–10 points	5.37^a^ (1–10)	5.76^a^ (1–10)
Sleep time, hours/day	9.87^a^ (8.19–11.8)	9.77^a^ (8.49–10.6)
Age, years	8.64^a^ (3.75–14.75)	9.31^a^ (5.4–13.83)
Height SDS, m	0.32^a^ (−1.78 to 2.71)	0.90^b^ (−1.26 to 2.54)
Body mass index SDS, Km/m^2^	−0.44^a^ (−2.58 to 1.88)	−0.34^a^ (−1.34 to 0.77)
Muscular mass, Kg	23.7^a^ (13.4–47.6)	29.7^b^ (16.5–55.9)
Fat mass, Kg	7.01 ^a^ (2–14)	6.62^a^ (3.3–17.5)
Right calf circumference, cm	26.9^a^ (24.5–34.5)	30.1^a^ (24.6–38)
Left calf circumference, cm	27.1^a^ (25–33.5)	28.5^a^ (24–32)
Right‐hand strength, Kg	14.6^a^ (4–25)	18.6^a^ (9–39)
Left hand strength, Kg	14.4^a^ (4–23)	17.4^b^ (7–32)
Flexibility, cm	27.9^a^ (10–37.5)	22.3^a^ (14–33)
Biceps brachii ROM	108.3^a^ (95–208)	100.2^a^ (14–115)
Pronator teres ROM	117.7^a^ (90–164)	120.2^a^ (90–150)
Adductor longus ROM	49.4^a^ (28–100)	90.8^b^ (18–196)
Lateral gastrocnemius and soleus ROM	32.4^a^ (18–73)	30.4^a^ (10–54)

*Note:* a, b: Estimates with the same letter in the superscript did not statistically differ with *p*‐value > 0.05 (*U* Mann–Whitney test).

Abbreviations: MVPA, moderate‐to‐vigorous physical activity; ROM, range of motion; SDS, standard deviation score.

### 2.1 Sample Size Justification

An a priori sample size justification was performed based on expected variability in SWE measurements and effect sizes reported in previous pediatric studies. A sample of 44 TD children was considered sufficient to detect clinically meaningful differences and associations in muscle elastic properties within an exploratory framework.

### 2.2 Statistical Analysis

All analyses were conducted using IBM® SPSS® Statistics software (version 19.0, International Business Machines Corporation, Armonk, NY, USA). Intra‐operator reliability for SWE measurements was evaluated by the ICC for each muscle at rest and MPS. To illustrate relationships among the SWE of all different muscles, a principal components analysis was performed at rest and MPS. Principal components explaining more than 10% of variance were retained. Analyses focused on the characterization of SWE values, and relationships between SWE and healthy habits were performed for each muscle in both positions. The general model accounted for gender as discrete factor, and continuous covariates consistent in perinatological antecedents—including gestational age, birth weight and birth length—, anthropometry—including weight, height, body mass index (BMI), waist, calf circumference, muscle and fat mass—and physical fitness—including upper‐body muscular strength, flexibility and ROM—and healthy habits—including MD, moderate‐to‐vigorous physical activity (MVPA) and sleep time—as linear and quadratic factors. First‐degree interactions between all factors were also evaluated. This model was refined by the stepwise variable selection algorithm that iteratively included (or discarded) significant (nonsignificant) factors and evaluated the goodness of fit of the hierarchical structure using standard statistical criteria. Significance was set to *p* < 0.05. The Shapiro‐Wilk test was used to corroborate normality of SWE residuals (*p* > 0.05) once fit the models described below. Age‐stratified analyses were additionally performed to explore potential age‐related differences in SWE values. No missing data were observed for primary SWE outcomes.

All analyses were considered exploratory and hypothesis‐generating. Covariates were selected a priori based on physiological plausibility and previous pediatric musculoskeletal literature, rather than for predictive modeling purposes. Normality of SWE variables was assessed using the Shapiro–Wilk test prior to modeling, and normality of residuals was confirmed after model fitting. No formal correction for multiple comparisons was applied, and results should therefore be interpreted with caution.

## 3 Results

SWE reliability and dimensional structure were assessed. Intra‐operator ICC for SWE is provided in Table 2. All estimates exceeded 0.95 at rest and 0.87 at MPS, with *p*‐value < 0.01. The highest ICCs were obtained in the AL muscles at both positions and in PT and the SOL at rest, although differences among muscles were small.

**Table 2:** Intraclass correlation coefficient of shear wave elastography measurements for each muscle at rest and during maximum passive stretching. Values are reported as intraclass correlation coefficients with 95% confidence intervals.

	Intraclass correlation coefficient	Confidence interval 95%
Rest		
Biceps brachii	0.96^a^	0.93–0.95
Pronator teres	0.97^b,c^	0.96–0.98
Adductor longus	0.98^b^	0.97–0.98
Lateral gastrocnemius	0.95^a,c^	0.93–0.96
Soleus	0.97^b,c^	0.95–0.98
Maximum passive stretching		
Biceps brachii	0.91^a,b^	0.87–0.94
Pronator teres	0.87^a^	0.81–0.9
Adductor longus	0.95^b^	0.94–0.97
Lateral gastrocnemius	0.91^a,c^	0.87–0.93
Soleus	0.92^a,c^	0.89–0.94

*Note:* a, b, c: Estimates with the same letter in the supper‐script did not statistically differ with (*p* > 0.05).

**Figure 1:**
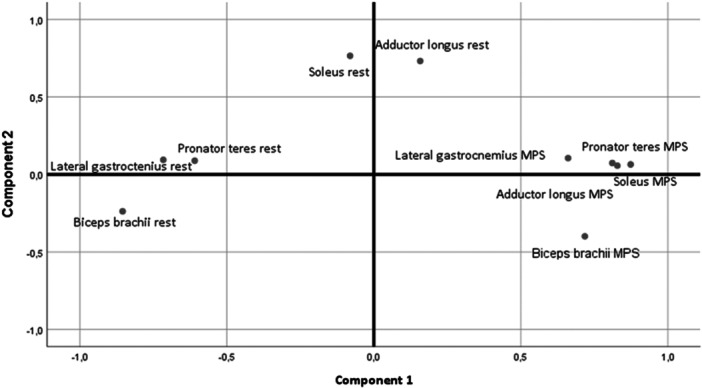
Main component analysis results for each muscle at rest and maximum passive stretching. MPS, maximum passive stretching.

Principal component analysis of SWE data revealed two main components explaining 47.1% and 13.8% of total variance. The next three components explained 9.5%, 7.1%, and 6.3%, while the rest contributed less than 5%. The first component primarily separated muscles by joint position, with lower discrimination for AL and SOL at rest. The second component differentiated Al and SOL at rest from other muscles. Overall, the analysis grouped data into three clusters: All muscles at MPS position, AL and SOL muscles at rest, and BB, PT, and LG at rest (Figure 1).

**Figure 2:**
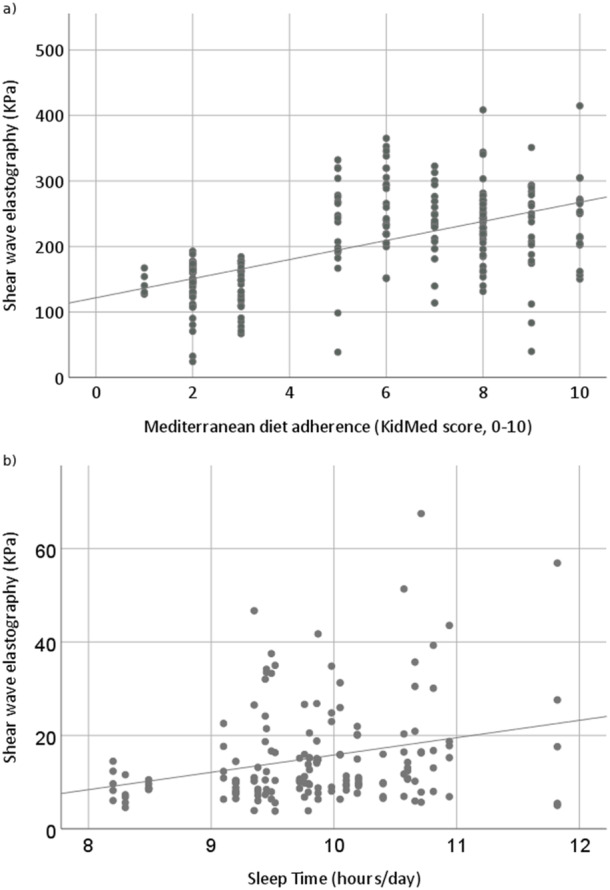
Association between of healthy habits and shear wave elastography (SWE) values in typically developing children. (a) Relationship between adherence to the Mediterranean diet (KidMed score, range 0–10) and SWE values (kPa) measured during maximum passive stretching across the evaluated muscles. (b) Relationship between sleep duration (hours/day) and SWE values (kPa) measured at rest.

SWE values were analyzed in relation to joint position, muscle group, and lifestyle factors. Average SWE values are shown in Table 3 and the distribution of measurements across muscles and joint positions is presented in Supplementary Figure S1. SWE values were significantly higher at MPS than at rest across muscles. Despite slightly higher SWE at rest in upper limb muscles, no differences were found between muscles within the same position. No significant associations were observed with demographic, anthropometric, or physical fitness variables. However, healthy habits (i.e., MD adherence, MVPA, and sleep time) had significant effects on SWE (Table 4). Higher MD adherence increased SWE at MPS across all muscles (Figure 2a), while a decrease was observed at rest in BB and PT. SWE at rest increased with sleep time in all muscles (Figure 2b), with a slight decrease at MPS only in AL. Finally, MVPA was associated with a small but significant increase in SWE in the PT muscle at rest and AL at MPS (Table 4).

**Table 3:** Shear wave elastography values of the evaluated muscles in typically developing children at rest and during maximum passive stretching. Values are expressed in kilopascals (kPa) as mean ± standard deviation.

	Rest	Maximum passive stretching
Biceps brachii	21.4 ^a^ ± 14.9	187.4 ^b^ ± 9.01
Pronator teres	18.2 ^a^ ± 9.17	233.7 ^b^ ± 37.3
Adductor longus	11.7 ^a^ ± 6.23	176.3 ^b^ ± 53.1
Lateral gastrocnemius	12.4 ^a^ ± 4.94	199.1 ^b^ ± 45.7
Soleus	10.1 ^a^ ± 4.91	198.4 ^b^ ± 45.7

*Note:* a, b: Estimates with the same letter in the superscript did not statistically differ with *p*‐ > 0.05.

**Table 4:** Association between healthy habits factors and shear wave elastography values in typically developing children for each muscle at rest and during maximum passive stretching. Results are reported as regression coefficients (B) with standard errors and corresponding *p*‐values.

	Adherence to the Mediterranean diet		Moderate to vigorous physical activity		Sleep time	
Rest						
Biceps brachii	**−3.66** ± **0.57**	(0.04)	−0.06 ± 0.05	(NS)	**7.40** ± **1.14**	(0.03)
Pronator teres	**−1.4** ± **0.56**	(0.02)	**0.14** ± **0.05**	(0.03)	**2.97** ± **1.24**	(0.02)
Adductor longus	0.4 ± 0.65	(NS)	0.04 ± 0.05	(NS)	**2.32** ± **1.08**	(< 0.01)
Lateral gastrocnemius	−0.96 ± 0.57	(NS)	0.04 ± 0.05	(NS)	**3.04** ± **1.03**	(0.04)
Soleus	0.16 ± 0.57	(NS)	0.04 ± 0.05	(NS)	**2.16** ± **1.03**	(< 0.01)
Maximum passive stretching						
Biceps brachii	**16.7** ± **3.97**	(< 0.01)	−0.04 ± 0.32	(NS)	−0.48 ± 7.9	NS
Pronator teres	**18.1** ± **3.95**	(< 0.01)	0.24 ± 0.31	(NS)	−6.03 ± 8.62	NS
Adductor longus	**17.4** ± **4.49**	(< 0.01)	**0.73** ± **0.31**	(0.03)	**−12.76** ± **7.53**	(0.03)
Lateral gastrocnemius	**15.5** ± **3.94**	(< 0.01)	0.4 ± 0.31	(NS)	−5.7 ± 7.14	NS
Soleus	**25.4** ± **3.93**	(< 0.01)	0.28 ± 0.31	(NS)	−7.2 ± 7.14	NS

*Note:* Results are shown as *B*‐value ± deviation error (*p‐*value) from general linear model at rest (*p*‐value < 0.05; *R*‐square: 0.67); and from general linear model at MPS (*p*‐value < 0.05; *R*‐square: 0.61). Estimates in bold type were statistically significant. Nonstatistically significant variables in the general linear model: gender (years), gestational age (weeks), birth weight (g), birth length (cm), weight (SDS), height (SDS), body mass index (SDS), waist (cm), calf circumference (cm), muscle mass (Kg), fat mass (Kg), right and left upper‐body muscular strength (Kg), flexibility (cm), joint range of motion (degrees).

Abbreviation: NS, nonstatistically significant.

## 4 Discussion

Our findings demonstrate that SWE provides good to excellent reliability for all evaluated muscles, both at rest and during MPS. Additionally, the principal component analysis identified two major components, resulting in the delineation of three distinct muscle groups. In our pediatric population, healthy lifestyle habits influenced SWE at rest and MPS, with variable patterns and intensities.

In assessing SWE reliability, our protocol focused on the dominant side of each child, a decision grounded in literature on muscle elasticity. While Berko et al. [24] reported increased elasticity in the dominant BB via strain elastography, other studies found no side differences in LG and rectus femoris using SWE and ARFI elastography [2, 33]. Notably, no previous SWE studies have systematically assessed both limbs in the upper extremity. Therefore, focusing on the dominant side is considered sufficient to characterize SWE in TD children and is more time‐efficient for clinical implementation.

To ensure measurement reliability, five repeated SWE scans were obtained for each muscle in both rest and MPS positions. Intra‐operator reliability was assessed by the ICC, consistent with methodology in the literature [27, 34]. All ICC estimates exceeded 0.86 and were statistically significant, indicating excellent reliability according to Koo and Li's [35] criteria (> 0.9), except for the PT muscle at MPS position, which fell within good reliability (0.7–0.9) [35]; see Table 3. These findings align with adult SWE studies [36, 37] and surpass previous pediatric data [3, 23, 38, 39]. The improved ICC values may reflect our flexible approach to joint angle positioning, allowing individualizes adjustment rather than imposing a standardized angle. Therefore, neither the young age of participants nor intermuscle variability negatively impacted measurement reliability. SWE thus appears to be a robust method for quantifying muscle elasticity in children, at rest and MPS, supporting its applicability in further analyses.

Our findings are consistent with previous adult studies demonstrating that SWE is sensitive to changes in passive muscle stiffness under different mechanical or therapeutic conditions [18]. From a biomechanical perspective, passive muscle stiffness reflects the combined contribution of intrinsic muscle tissue properties, connective tissue components, and joint positioning, which may explain the attenuation of intermuscle differences observed during MPS.

Principal component analysis revealed two major components that accounted for more than 10% of total variability each. When muscles were plotted based on these two components, three main clusters emerged, including all muscles at MPS, the AL and SOL muscles at rest, and PT, BB, and LG muscles at rest. These results suggested that intrinsic differences between muscles become attenuated in the MPS position, whereas they remain more pronounced at rest. It is important to note that AL and SOL muscles cross a single joint in the lower limb, while the PT, BB, and LG muscles span at least two joints in the upper and lower limbs. Only the primary joint was considered for defining the rest; consequently, secondary joints and associated anatomical structures may have influenced results. Additionally, joint position, muscle architecture, and probe orientation are recognized factors influencing SWE measurements in skeletal muscle studies [13, 22]. This muscle grouping pattern does not compromise previous ICC estimates, as discussed below. These results should guide future SWE studies to include representative muscles from each subgroup. From a clinical perspective, this dimensional structure suggests that muscle elastic behavior clusters primarily according to joint position rather than anatomical location alone, which may have implications for the interpretation of SWE findings in pediatric assessment. The attenuation of intermuscle differences during MPS supports the concept of a shared biomechanical response under stretch conditions, potentially relevant for standardizing clinical SWE protocols.

Among our children population, adherence to the MD, MVPA time, and sleep time influenced SWE measurements at both joint positions, although with a certain degree of heterogeneity. While SWE values increased with higher MD adherence under MPS across all muscles, an inverse pattern was observed at rest, but only for the BB and PT muscles. Similarly, an increase in sleep time was associated with reduced SWE values at rest in all muscles, and with increased SWE only in the AL during MPS. Although MVPA yielded some significant associations, no consistent pattern emerged across muscles or joint positions (Table 4). The direction of these associations should be interpreted in light of joint‐specific mechanical behavior. Increased SWE values during MPS may reflect greater passive tension under elongation, whereas lower SWE values at rest could indicate reduced basal muscle tone. The coexistence of opposite patterns across joint positions, therefore, likely reflects differences between resting viscoelastic properties and stretch‐induced passive resistance rather than contradictory physiological effects.

These findings suggest that muscle elastic properties may be modified by structural changes in muscle tissue induced by lifestyle factors, particularly diet and sleep, although potential effects from physical activity cannot be entirely ruled out. This aligns with the notion that muscle elasticity may undergo long‐term adaptations in tissue composition, including collagen distribution and muscle fiber density [26], even in the absence of significant changes in muscle mass. Although several associations reached statistical significance, the magnitude of the observed effects was modest. Therefore, these findings should be interpreted cautiously, as small effect sizes may not necessarily translate into clinically meaningful differences at the individual level. However, even subtle changes in passive muscle mechanical properties during childhood could reflect early physiological adaptations to lifestyle behaviors and may acquire greater relevance over time or at the population level.

Recent studies demonstrated improvements in physical and mental health [13, 14] as well as strength in children [14] and young adults [15] associated with adherence to MD, albeit with varying effects depending on adherence levels. In our study, children with higher MD adherence demonstrated increase SWE values at MPS, which may indicate greater muscular resistance to stretching, unrelated to strength, flexibility, or joint ROM. This increase in stiffness during MPS may suggest an enhanced ability to tolerate elongation, and thus, a potential protective mechanism against overstretch‐related injuries. Conversely, we observed a decrease in SWE at rest associated with MD adherence in two upper limb muscles, possibly reflecting greater muscle relaxation under basal conditions, a feature that could be linked to improved physiological elasticity and neuromuscular control. It is important to highlight that MD adherence has a deep impact on obesity [40, 41], which in turn may lead to changes in muscle composition detectable through SWE. The lack of significance in lower limb muscles findings does not invalidate these interpretations, as dietary effects likely vary based on muscle architecture, length, and volume. Interestingly, even in the LG muscle—which did not reach statistical significance—the trend was consistent with the upper limb muscles, suggesting a potentially similar underlying mechanism. Larger studies are warranted to explore these hypotheses further.

We also found that children who slept longer exhibited increased muscular elasticity at rest. Longer sleep time, especially when combined with higher MVPA, has been linked to lower fat mass index, reduced metabolic syndrome risk, enhanced musculoskeletal fitness, and improved bone health [5, 9]. Current guidelines recommend 9–12 h of sleep per night for school‐aged children (6–12 years) [8]. Notably, most participants met these recommendations, and variation was narrow. Despite this, we detected a significant association between longer sleep time and improved muscle relaxation at rest. This may reflect more effective recovery processes and hormonal regulation impacting muscle tone. On the other hand, results related to MVPA were inconsistent, with statistically significant effects limited to two muscles of upper and lower limb each, and across different joint positions. As physical activity enhances strength and endurance without altering tissue structure [9], the lack of consistent MVPA on SWE appears reasonable. Collectively, these findings suggest that dietary and sleep habits may exert a more decisive influence on muscle structural adaptations than physical activity during this developmental stage.

Although some studies have reported significant differences between adult men and women [42], such gender and age effects have not been consistently confirmed in children [3, 16, 21, 24]. This is consistent with our findings, where SWE did not differ by gender or age. Historically, BMI has been used as a general marker of body mass composition, and calf circumference as a proxy for muscle mass in elastography studies, although results have been inconsistent across muscles and populations [3, 24]. Even with similar BMI or calf size, individual differences in tissue compositions may influence passive muscle elasticity [3]. To improve precision, we incorporated body composition analysis via bioelectrical impedance. Nevertheless, our findings did not reveal significant effects on muscle mass, as measured by calf circumference or bioimpedance, on SWE values.

Passive muscle elasticity, alongside strength and joint ROM, is a relevant factor in predicting physical function [43], although interactions with these variables remain incompletely understood. While muscle strength has been associated with muscle elasticity [43], our analyses using hand grip strength data did not report significant effects on SWE. Moreover, although not statistically significant, Brandenburg et al. [3] reported a negative association between passive maximal ankle dorsiflexion and passive LG muscle elasticity measured via SWE. They attributed this to the isolated analysis of LG muscle without accounting for SOL muscle contribution. In our case, LG and SOL muscles were evaluated together, as they both influence passive ankle dorsiflexion. Other anthropometric and physical fitness characteristics showed no influence.

It is worth noting that in children and adolescents, muscle development—including increases in size and strength—continues into the early twenties. Within this context, nutrition and sleep may act as essential modulators of muscular development and passive biomechanical properties.

Finally, based on our results, we propose that increased muscle stiffness during passive stretch (reflected by higher SWE at MPS) may indicate a more resilient muscular structure, capable of resisting deformation. In contrast, lower SWE at rest may denote better basal muscle relaxation and recovery capacity. These properties, potentially influenced by dietary and sleep behaviors, could contribute to enhanced postural control, reduced risk of injury, and more efficient movement patterns. Importantly, given the cross‐sectional and observational design of the study, causal inferences cannot be established. The associations observed between MD adherence, sleep duration, and SWE values should therefore be interpreted as correlational findings rather than evidence of a direct causal relationship. Larger longitudinal studies are required to determine whether healthy lifestyle behaviors exert a causal influence on passive muscle mechanical properties during childhood. Future research should aim to validate these interpretations in larger pediatric cohorts.

## 5 Limitations and Strengths

Several limitations should be acknowledged. First, for biarticular muscles, no data were collected regarding the secondary joint, which may have influenced resting muscle tension and SWE values. Second, measurements were limited to the dominant side, which restricts the interpretation of potential bilateral differences. Third, although an a priori sample size justification was performed, the relatively small sample size and the number of variables analyzed increase the risk of overfitting: consequently, the findings should be interpreted as exploratory. In addition, participants were recruited from a single geographic area, which may limit the generalizability of the results to other pediatric populations.

The study also has notable strengths. We included five muscles—two from the upper limb and three from the lower limb—selected based on their functional relevance and patterns of motor involvement in pediatric populations. This extended muscle set provides a broader and clinically meaningful reference for SWE studies in children. A further strength is the inclusion of healthy habits not evaluated in previous muscle elasticity research studies.

## 6 Conclusions

Our study supports the use of SWE as a valid, objective, and reliable tool for assessing muscle properties in TD children. Furthermore, it suggests that SWE is sensitive enough to detect measurable variations in muscle elasticity associated with lifestyle factors, particularly nutrition and sleep time. These findings highlight the potential of SWE as a noninvasive tool to quantify the impact of healthy habits on muscle structure and possibly on broader aspects of physical function. However, further research with larger pediatric cohorts is required to confirm these associations and to explore their long‐term functional significance.

## Author Contributions

**Dolors Casellas‐Vidal:** conceptualization, investigation, writing – original draft, methodology, writing – review and editing, formal analysis. **Raquel Font‐Lladó:** writing – review and editing, investigation. **Inés Osiniri:** writing – review and editing, investigation, methodology. **Maria Camós‐Carreras:** investigation, writing – review and editing, conceptualization. **Aintzane Ruiz‐Eizmendi:** writing – review and editing, methodology, investigation. **Juan Serrano‐Ferrer:** writing – review and editing, investigation. **Joaquim Casellas:** formal analysis, writing – review and editing, methodology, supervision. **Abel López‐Bermejo:** writing – review and editing, supervision. **Anna Prats‐Puig:** investigation, methodology, writing – review and editing.

## Ethics Statement

We confirm that we have read the Journal's position on issues involved in ethical publication and affirm that this report is consistent with those guidelines. This study was approved by the Ethics Committee of the *Hospital Universitari de Girona Dr. Josep Trueta* (CEIC V4_14/11/2016). The research was conducted in full accordance with the ethical standards set forth by the Declaration of Helsinki and the institution's guidelines for research involving human subjects. All participants were provided with detailed information regarding the purpose of the study, procedures involved, and their rights as participants. Written informed consent was obtained from all participants before their inclusion in the study. Participation was voluntary, and participants had the right to withdraw from the study at any time without penalty. Confidentiality and anonymity were ensured throughout the research process, and all collected data were de‐identified and securely stored.

## Conflicts of Interest

The authors declare no conflicts of interest.

## Transparency Statement

The corresponding author affirms that this manuscript is an honest, accurate, and transparent account of the study being reported. All authors confirm that the journal's policies and ethical guidelines have been reviewed and fully complied with, and that the manuscript is original, not published elsewhere, and not under consideration by another journal.

## Supporting information


**Figure S1:** Distribution of shear wave elastography (SWE) values (kPa) at rest and during maximum passive stretching (MPS) in typically developing children. Boxplots represent median, interquartile range, and outliers. AL, adductor longus; BB, biceps brachii; GL, lateral gastrocnemius; MPS, maximum passive stretching; PT, pronator teres; SOL, soleus.

## Data Availability

The datasets generated during and/or analyzed during the current study are available from the corresponding author on reasonable request.
