# Supplementary figures and images for: Shear Wave Elastography in Children: Normative Values and the Impact of Healthy Habits in a Cross‐Sectional Study
Source: Health Sci Rep. 2026 Jun 19;9(6):e72653. doi: 10.1002/hsr2.72653 (PMC13281414; doi:10.1002/hsr2.72653)

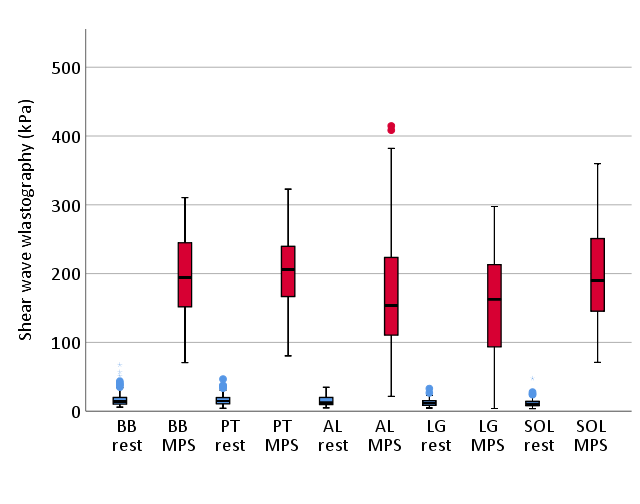

Supplement: Supplementary file 1 — Figure S1: Distribution of shear wave elastography (SWE) values (kPa) at rest and during maximum passive stretching (MPS) in typically developing children. Boxplots represent median, interquartile range, and outliers. AL, adductor longus; BB, biceps brachii; GL, lateral gastrocnemius; MPS, maximum passive stretching; PT, pronator teres; SOL, soleus. [file HSR2-9-e72653-s001.png]
